# Jiaji (EX-B2)-Based Electroacupuncture Preconditioning Attenuates Early Ischaemia Reperfusion Injury in the Rat Myocardium

**DOI:** 10.1155/2020/8854033

**Published:** 2020-12-08

**Authors:** Ning Xiao, Yang Li, Ming-lu Shao, Hua-feng Cui, Chang-yun Zhang, Su-ping Kong, Xin Zhang, Hui-juan Yu, Qi-wen Tan

**Affiliations:** ^1^College of Acupuncture and Moxibustion, Shandong University of Traditional Chinese Medicine, Jinan 250014, China; ^2^Shanghai Pudong People's Hospital, Shanghai 201299, China; ^3^Hospital Affiliated to Shandong University of Traditional Chinese Medicine, Jinan 250014, China

## Abstract

**Background:**

Acupuncture preconditioning was able to reduce the extent of ischaemia reperfusion (*I*/*R*) injury. Previous studies have shown that electroacupuncture (EA) pretreatment at T4-T5 Jiaji (EX-B2) acupoints had cardioprotective effects against myocardial *I*/*R* injury. However, the molecular mechanism remains inconclusive.

**Methods:**

Wistar rats were pretreated with electroacupuncture for 7 days at the Neiguan (PC6), T4-T5 Jiaji (EX-B2), Yanglingquan (GB34), and Quchi (LI11) acupoints, which belong to different meridians. Then, we investigated the genome-wide gene expression profiles of rats prestimulated at these acupoints after *I*/*R* injury.

**Results:**

Our study revealed previously unknown cardioprotective roles of T4-T5 Jiaji (EX-B2) acupoints in the *I*/*R* progression. The extent of myocardial injury was significantly decreased in the Jiaji group compared with the *I*/*R* group. In addition, our data are among the first to link the EA preconditioning at Neiguan (PC6) acupoints and circadian rhythm in the *I*/*R* model. Also, for the first time, we explored the meridian and acupoint specificity involved in EA pretreatment at the heart meridian, in which Yanglingquan and Quchi acupoints were selected as the control group for heart-divergent-meridian and nonheart-meridian acupoints.

**Conclusions:**

The present study suggested that EA pretreatment at Jiaji alters genome-wide gene expression and protects the rat myocardium against *I*/*R* injury, which are most likely through neurohumoral regulation.

## 1. Introduction

Growing evidence has shown that acupuncture has therapeutic effects on certain types of hypertension, angina pectoris, coronary heart disease, arrhythmia, and ischemic heart disease [1–4]. According to traditional Chinese medicine (TCM) theory, different acupoints belong to different meridians and are associated with different Zangfu-organs. The specificity of acupoints for the corresponding Zangfu-organ has a neurobiological basis. The acupoint Neiguan (PC6), on the pericardium meridian, has been shown to function in cardiac protection. It has been documented that electroacupuncture (EA) pretreatment at PC6 could improve heart function and energy metabolism, promote angiogenesis after ischaemia [5, 6], reduce cardiac troponin I (cTnI) release [7], and protect against myocardial injury and has an antiarrhythmic effect [4]. Numerous studies have shown the protective mechanism of PC6 in animal models of myocardial ischaemia reperfusion (*I*/*R*) injury [8–11].

The Jiaji (EX-B2) acupoints, which are closely related to the Zangfu-organ, are extra nerve acupoints adjacent to dorsal-shu points. EX-B2 acupoints are sensory points that reflect the pathophysiology of the Zangfu-organ. Therefore, EX-B2 acupoints are often used to treat the corresponding Zangfu-organ disease in clinical practice more safely and effectively than treatment with dorsal-shu points. The T4-T5 Jiaji (EX-B2) acupoints correspond to Jueyinshu (BL14), which is the dorsal-shu point of the pericardium meridian, and Xinshu (BL15), which is the dorsal-shu point of the heart meridian. Evidence from clinical practice has shown that acupuncture at T4-T5 Jiaji (EX-B2) acupoints can improve angina pectoris and promote myocardial ischaemia tolerance in adult patients [12]. In addition, our previous experimental findings also suggested that EA at T4-T5 Jiaji (EX-B2) acupoints play a protective role against cardiac *I*/*R* injury by modulating apoptosis gene expression [13]. We also observed that EA at the T4-T5 Jiaji (EX-B2) acupoint increases nitric oxide (NO) release and attenuates endothelin (ET) and proinflammatory interleukin-1 beta (IL-1*β*) expression [14]. However, the details of the protective mechanisms of EA at the T4-T5 Jiaji (EX-B2) acupoints have not been fully elucidated. Moreover, the specificity of acupoints and meridians is not clearly understood.

Acupuncture at the Yanglingquan (GB34) acupoint, which belongs to the gallbladder meridian, has been reported to increase the blood flow velocity of the anterior cerebral artery and to have a good curative effect on nervous system disease [15, 16]. Although GB34 is not an acupoint that is directly related to the heart, it contacts the heart with its divergent meridians (branches of the regular meridians). The Quchi acupoint (LI11), which is located on the large intestinal meridian, is neither directly associated with the heart nor indirectly connected to the heart by divergent meridians. In the clinic, LI11 has been applied to cure digestive system diseases and hypertension [17, 18].

Numerous studies have reported the protective mechanism of EA at Neiguan (PC6), but there has been no report about the mechanism of acupuncture at Jiaji (EX-B2) acupoints during the *I*/*R* process. In this study, we intended to characterize the gene expression profiles of electroacupuncture preconditioning at T4-T5 EX-B2 acupoints in myocardial *I*/*R* rats by microarray analysis and with respect to the specificity of acupoint electroacupuncture preconditioning at different acupoints. Therefore, we stimulated the PC6, T4-T5 EX-B2, GB34, and LI11 points with EA preconditioning. Our data indicate that EA at T4-T5 EX-B2 acupoints has protective effects on the myocardium. In addition, in the present study, we highlight distinct gene expression profiles across different acupoints in *I*/*R* rats. The different acupoints not only showed specific characteristic gene expression but also overlapped in some signaling pathways.

## 2. Materials and Methods

### 2.1. Animal

This study conformed to the Guide for the Care and Use of Laboratory Animals China and was approved by the Animal Care Committee of Shandong University of Traditional Chinese Medicine (*SDUTCM20150312001*). Commercially available Wistar rats (200 g–250 g) were purchased from Shandong Lukang Animal Ltd., Co. (Jining, China). All animals were housed in a conventional housing facility under a standard 12 : 12 h light-dark cycle. The rats received standard diet and water ad libitum and were given 1 week to accommodate. Animals were randomly selected from the group-housed animals in each cage and assigned to different experimental groups. An investigator blinded to the experiment was assigned the task of administering treatments and distributing animals into experimental groups.

### 2.2. Electroacupuncture Preconditioning

**Figure 1:**
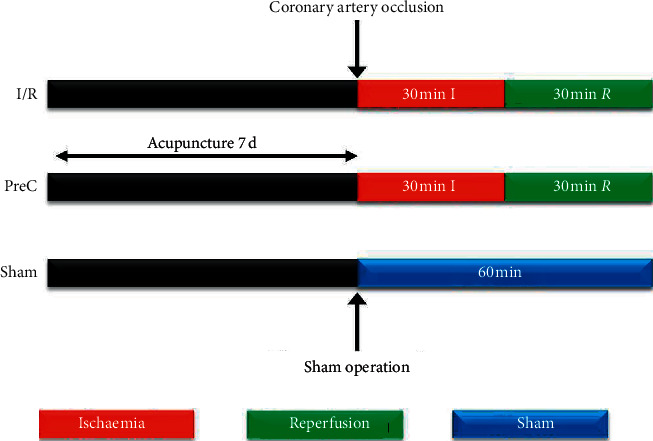
Experimental workflow. *I*/*R* : ischaemia reperfusion group; PreC : electroacupuncture preconditioning group; Sham: sham operation group.

After adaptive feeding for one week, male and female (*n* = 60, respectively) rats were randomly divided into six groups: the sham operation control group (Sham), myocardial ischaemia reperfusion model group (*I*/*R*), EA at EX-B2 T4-5 + *I*/*R* (JJ), EA at PC6 + *I*/*R* (NG), EA at GB34 + *I*/*R* (YLQ), and EA at LI11 + *I*/*R* (QC). Rats in the JJ, NG, YLQ, and QC groups were pretreated with electroacupuncture for 7 days before induction of ischaemia or tissue harvesting. Electrical current was provided to the needles through an electrical stimulator with a stimulus isolation unit (Han Acuten, WQ1002F, Beijing, China). An acupuncture needle, 7 mm long and 0.16 mm in diameter, was inserted 2-3 mm into the subcutis. The parameters of EA were a pulse width of 0.3 ms, alternating frequency of 2 Hz and 15 Hz, and intensity level of 1-2 mA, for a total stimulation period of 30 minutes. The rationale for using alternating frequencies (2–15 Hz) in animals is to avoid the development of nervous stimulation tolerance. The timeline of the study is shown in Figure 1.

### 2.3. IR Models

Rats were anaesthetized by isoflurane inhalation, and body temperature was maintained on a heating pad (37°C). All rats were subjected to ischaemia and reperfusion of the heart as described previously [19]. After 30 min occlusion of the left anterior descending coronary artery (LAD), the heart was allowed to reperfuse. The sham operation animals underwent the same procedure without occlusion of the LAD.

### 2.4. Histopathology and Immunohistochemistry (IHC)

Rats were anaesthetized with an intraperitoneal injection of sodium pentobarbital (50 mg/kg) and then euthanized by cervical dislocation. The left ventricles were harvested, fixed in 4% PFA, and embedded in paraffin. Sections (4 *μ*m) were stained with haematoxylin and eosin (HE) or Masson's trichrome according to the manufacturer's protocols (Sigma-Aldrich).

Immunohistochemistry staining was performed on paraffin-embedded slices as described above. The antibodies used in this study were *α*-smooth muscle actin (*α*-SMA; ab7817, Abcam; 1 : 200) and CD45 (ab10558, Abcam; 1 : 800). For all immunohistochemistry staining, images were captured under a Leica microscope with microscope image-acquisition software.

### 2.5. Microarray Expression Profiling

Genome-wide transcriptional profiling with an Agilent microarray was carried out to identify genes whose expression may have been altered and involved in IRI. Briefly, total RNAs were extracted, and RNA samples extracted from rats subjected to Sham (*n* = 12), *I*/*R* (*n* = 12), JJ (*n* = 12), NG (*n* = 12), YLQ (*n* = 12), or QC (*n* = 12) treatment were subpooled in 4 mixtures. After quality control, RNAs were subjected to the Rat 4 × 44 K Gene Expression Array according to the manufacturer's instructions (Agilent). The scanning procedure was performed using an Agilent Microarray Scanner (Agilent G2565BA) at the KangCheng Bio-Tech Corporation in Shanghai, China. Agilent Feature Extraction Software was used for data analysis. Differentially expressed genes (DEGs) with statistical significance were identified through volcano plot filtering and fold change filtering. Adjusted *P* value < 0.05 and FoldChange ≥2 or FoldChange ≤0.5 were set as the thresholds for DEGs. Hierarchical clustering was performed using *R* scripts. GO analysis and pathway analysis were performed using the standard enrichment computation methods. All functions for enrichment analysis used the BH *P* value correction method, and GO terms and Kyoto Encyclopedia of Genes and Genomes (KEGG) pathways with a BH-adjusted *P* value <0.05 were considered statistically significant.

### 2.6. Bioinformatics Analysis

To dissect the relationships among the groups under the given conditions based on the microarray profiles, we applied Metascape analysis to visualize Gene Ontology (GO) functional enrichment results between multiple groups (https://metascape.org). The Metascape analysis was performed using the default settings. The GO chord plot was applied to obtain additional insights based on the fold changes in the JJ group. A heatmap was plotted by http://www.bioinformatics.com.cn, an online platform for data analysis and visualization. KEGG pathways were visualized with Cytoscape 3.5.1.

### 2.7. Statistical Analysis

The data are expressed as the means ± SDs. Comparisons between two groups were conducted by Student's *t*-test, whereas comparisons among 3 or more groups were conducted by ANOVA with Bonferroni correction for multiple comparisons. All data were analysed using GraphPad Prism 7.0 software and SPSS 19.0 software. A *P* value < 0.05 was considered statistically significant.

## 3. Results

### 3.1. EA at the Jiaji Acupoint Ameliorated Ischaemia Reperfusion Injury-Induced Myocardial Impairment

**Figure 2:**
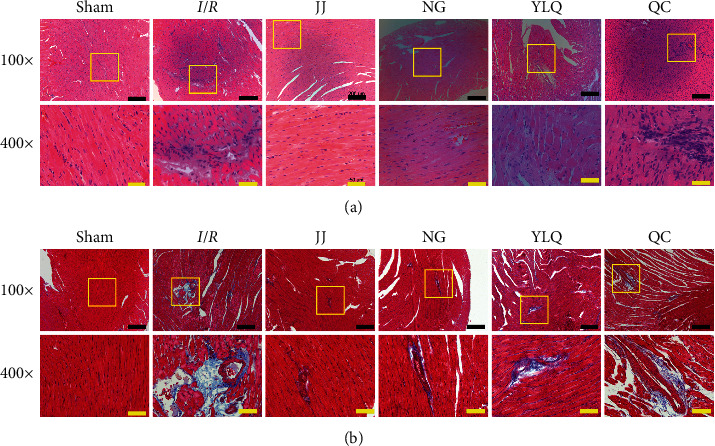
Histological characterization of EA preconditioning at T4-T5 EX-B2 ameliorated myocardial *I*/*R* injury. (a) Representative images of haematoxylin and eosin (HE) staining in different groups. (b) Representative images of Masson's trichrome staining from each group. The sections marked in rectangles are shown at greater magnification below. Scale bars: black, 200 *μ*m (100x); yellow, 50 *μ*m (400x).

To assess *I*/*R* injury, occlusion (30 min) of the left anterior descending coronary artery was followed by 30 min reperfusion. We did not find the myocardial infarction site in the *I*/*R* group or other EA preconditioning groups. This may be due to the short-term nature of the reperfusion, which occurs at the early stage of myocardial dysfunction. The extent of myocardial injury was assessed by HE and Masson's trichrome staining (Figures 2(a) and 2(b)). It was observed that myocardial cells and fibers maintained their structural integrity without inflammatory infiltration in the sham operation group. In the *I*/*R*, YLQ, and QC groups, the myocardial fibers were fractured and the myocardial cells showed edema and degeneration, as well as a small amount of necrosis, infiltration of inflammatory cells, and fibrosis. The JJ group, similar to the NG group, had significantly reduced cardiac tissue damage compared with the *I*/*R* group; the myocardial cell structure was preserved, with limited edema and degeneration. The degree of fibrosis appeared to be less dense in the JJ and NG groups. No significant differences were observed between the JJ and NG groups.

**Figure 3:**
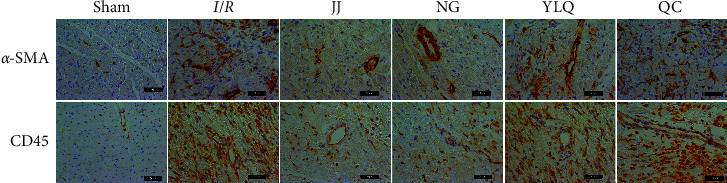
Representative images of *α*-SMA and CD45 immunohistochemistry in different groups. Upper panel: representative pictures of the immunohistochemistry stain for *α*-SMA expression in myocardial tissue to identify myofibroblasts. Bottom panel: representative CD45 immunohistochemistry to label inflammatory leukocyte infiltration in each group. Scale bars, 50 *μ*m.

Preconditioning EA at the Jiaji acupoint had relatively subtle effects on the early fibrotic response and inflammatory cell infiltration. To further assess this finding, we performed immunohistochemistry for *α*-SMA, which was associated with an accentuated early fibrotic response, and CD45, a marker of leukocytes. As shown in Figure 3, in the Sham group, *α*-SMA was distributed only in the blood vessel walls, while in the *I*/*R* group, *α*-SMA was detected throughout the myocardial tissue. Decreased *α*-SMA distribution in the myocardial tissues was observed in the JJ and NG groups, whereas a large amount of *α*-SMA distribution was observed in the YLQ and QC groups. Compared to the Sham group, the *I*/*R* group exhibited a large amount of inflammatory cell infiltration (CD45+); the YLQ and QC groups showed pathological features similar to those of the *I*/*R* group. CD45+ leukocytes were fewer in the JJ and NG groups. These results demonstrate that EA at the Jiaji acupoint ameliorated early fibrotic response and inflammatory cell infiltration.

### 3.2. Preconditioning EA at the Jiaji Acupoint Altered Genome-Wide Gene Expression

After confirming the cardioprotective effect of EA at the Jiaji (EX-B2) acupoint for *I*/*R* injury, we further investigated its possible mechanisms by microarray. The differentially expressed genes (DEGs) are summarized in Table 1. Compared to the Sham group, 571 genes were differentially expressed in the *I*/*R* group; out of these 571 genes, 115 genes were downregulated and 456 genes were upregulated. JJ pretreatment downregulated 161 genes and upregulated 558 genes compared with the *I*/*R* group, whereas NG pretreatment downregulated 53 genes and upregulated 520 genes compared with the *I*/*R* group. Compared to the *I*/*R* group, the YLQ and QC group upregulated 229 genes and 336 genes and downregulated 361 genes and 150 genes, respectively.

**Figure 4:**
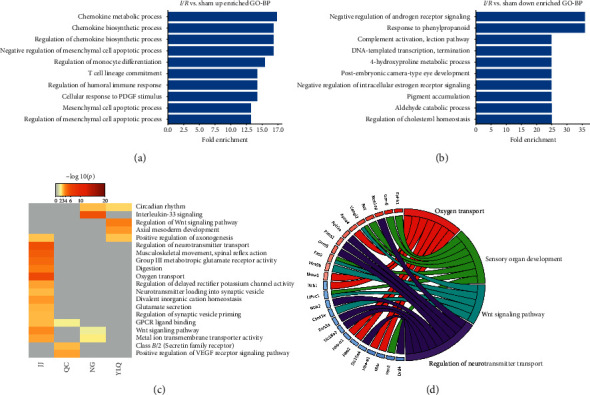
The GO biological processes (BPs) enriched among differentially expressed genes. The statistically significantly enriched BPs among upregulated genes (a) and downregulated genes (b) in the *I*/*R* group. (c)The heatmap for statistically significant enriched BPs by *P* values across groups compared to the *I*/*R* group. (d) GO Chord plot based on differentially expressed gene fold changes in the JJ group.

To investigate the functions of the differentially expressed genes, DEGs were subjected to disease/phenotype web-PAGE to identify potential biological process (BP) enrichment. The gene ontology (GO) analysis of the BP showed the fold enrichment value of the significant BP enrichment terms (Figure 4). The markedly increasing BPs in *I*/*R* vs. Sham were mainly related to immunity, chemokine, the early stage of inflammation-related factors, and cell apoptosis. Also, the negative regulation of sex hormones, DNA transcription termination, cholesterol homeostasis, and complement were significantly decreased (Figures 4(a) and 4(b)). All the EA preconditioning groups were compared to the *I*/*R* group, and the JJ group enriched more BPs than any other preconditioning groups. Neurohumoral regulation, metabolic processes, and immunity BPs were markedly increased in the JJ group. As shown in Figure 4(c), the BP most enriched in the JJ group according to *P* values was oxygen transport, and other enriched BPs were related to Wnt signal transduction and the neurotransmitter catabolic process, among others. The top fold changes showed similar results, indicating that EA at the Jiaji acupoint could improve oxygen transport (Figure 4(d)). Previous studies have revealed a strong correlation between interleukin-33 (IL-33) and myocardial fibrosis. Our data showed that the most important enriched BP in the NG group was IL-33 signaling (Figure 4(c)).

### 3.3. EA at the Jiaji Acupoint Showed a High Consistency with the Neiguan (PC6) Acupoint in Cardioprotective Effects

**Figure 5:**
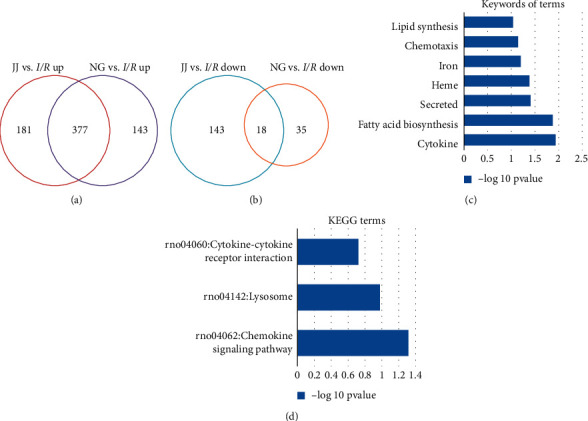
The consistency of significant differential gene expression (DEG) in the JJ and NG groups. (a) Venn diagrams of DEGs in the JJ and NG groups (vs. *I*/*R* group). Red and blue circles indicate the numbers of up- or downregulated genes in the JJ group (vs. *I*/*R* group); purple and yellow circles represent the numbers of up- or downregulated genes in the NG group (vs. *I*/*R* group). (b) Overlapping DEGs between the JJ and NG groups. (c) The keywords of GO and KEGG terms enriched among the 377 coupregulated genes. (d) KEGG terms.

The microarray results suggest that EA at the Jiaji (EX-B2) acupoint could alert the expression of different gene sets. To comprehensively elucidate the cardioprotective effects of the Jiaji acupoint, we analysed the similarities and differences in gene expression between Jiaji and Neiguan groups. Interestingly, EA at the Jiaji acupoint has a high consistency with the NG group in gene expression profiles. A total of 377 genes were coupregulated, and 18 genes were codownregulated (Figure 5(a)). The keywords of the co-DEGs were related to inflammation (*cytokine* and *chemotaxis*), steroid hormone biosynthesis (*fatty acid biosynthesis, secreted,* and *lipid synthesis*), and oxygen transport (heme and iron). The enriched KEGG pathways were the chemokine signaling pathway, lysosome, and cytokine-cytokine receptor interaction (Figure 5(b)).

### 3.4. Correlation Network Analysis of Differentially Expressed Genes

**Figure 6:**
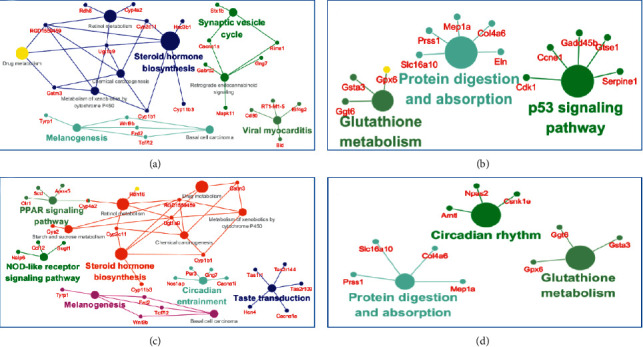
Visualization network of DEGs enrichment across groups. The KEGG analysis based on DEGs was performed with Cytoscape 3.5.1. To generate a correlation network, pathways are colored according to the taxonomic assignment as labeled, and the node size is scaled with the number of genes within the pathway. (a) JJ vs. *I*/*R* up. (b) JJ vs. *I*/*R* down. (c) NG vs. *I*/*R* up. (d) NG vs. *I*/*R* down.

To better understand the function of the DEGs, the correlation network was analyzed by KEGG pathway analysis with Cytoscape. All the pathways enriched across group comparisons are graphically displayed in Figure 6, and there were no GO terms found in other groups. EA preconditioning at Jiaji reversed the *I*/*R*-induced changes in some genes' expression. The JJ and NG groups both exhibited regulation of the steroid hormone biosynthesis and melanogenesis pathways. In parallel with the reduction in the p53 signaling pathway, there was a unique increase in the synaptic vesicle cycle and viral myocarditis terms in the JJ group (Figures 6(a) and 6(c)). The glutathione metabolism and protein digestion and absorption pathways declined in the JJ and NG groups (Figures 6(b) and 6(d)). These data indicate that EA at the Jiaji acupoint most likely exerted myocardial protection through neurohumoral regulation.

The unique upregulated pathways in the NG group were the PPAR signaling pathway and NOD-like receptor signaling pathway. Beyond that, we observed an interesting pathway that was uniquely alerted in the NG group: the expression of circadian rhythm-related genes decreased, whereas that of circadian entrainment-related genes increased (Figure 6(d)). Pathway analysis indicated that the antiarrhythmic effect of the Neiguan acupoint may be mediated by the regulation of circadian entrainment.

## 4. Discussion

To investigate the effects of electroacupuncture preconditioning at the T4-T5 Jiaji (EX-B2) acupoints, we first evaluated myocardial genome-wide transcriptional profiles across different acupoints in *I*/*R* rats. The results revealed previously unknown cardioprotective roles of the T4-T5 Jiaji (EX-B2) acupoints in *I*/*R* progression and provided a comprehensive view of the specificity of meridians and acupoints. In addition, our data are among the first to link EA preconditioning at Neiguan (PC6) acupoints and circadian rhythm in an *I*/*R* model.

Our previous studies had demonstrated that electroacupuncture at Jiaji (EX-B2) could effectively protect the myocardium from *I*/*R* injury [13, 14]. However, the molecular mechanism underlying the protective effects of acupuncture on Jiaji (EX-B2) remains unclear. The histopathology suggested that EA at Jiaji (EX-B2) significantly reduced cardiac tissue damage and led to less dense fibrosis. Then, we explored the genome-wide gene expression after EA preconditioning at Jiaji (EX-B2) acupoints. In the JJ group, the expression of genes related to inflammation (*cytokine* and *chemotaxis*) was comprehensively reduced, while that related to neurohumoral regulation-steroid hormone (*estrogen* and *testosterone*) biosynthesis was significantly increased. Both experimental and clinical evidence have suggested that steroid hormone-estrogen and testosterone protect against CHD and *I*/*R* [20–22]. Several studies have reported that electroacupuncture protects against *I*/*R* by modulating the autonomic nervous system's control of cardiac function. In addition, inhibiting the cardiac sympathetic nervous system could exert protective effects on the myocardium [11, 23]. Electroacupuncture pretreatments have been further described to play a critical role in stimulating sympathetic activity, inducing the desensitization of *β*-adrenergic receptors via a mechanism similar to that of ischemic preconditioning [9, 24]. In the present study, electroacupuncture at Jiaji (EX-B2) acupoints induced *γ*-aminobutyric acid (GABA) secretion, peripheral nervous system axon regeneration, and ganglion development. GABA is the major inhibitory neurotransmitter in the central nervous system and is involved in many metabolic activities. GABA can antagonize the neurotoxicity of glutamate (Glu) and play a protective role in cerebral ischemic injury. By reducing unconscious movement, increasing cerebral blood flow, and decreasing brain metabolism, energy consumption is reduced [25].

Neiguan (PC6) is on the pericardium meridian, which is the most important heart-related meridian in all 12 meridians. Numerous studies have reported that acupuncture at PC6 could reduce arrhythmias, apoptosis, and myocardial enzymes [11, 23, 24, 26] and inhibit autophagy [27]. Fu et al. had demonstrated that EA at PC6 could effectively promote angiogenesis and protect myocardial tissue against ischaemia injury [5]. Acupuncture pretreatment at PC6 protects heart via the vagal nerve and its nicotinic receptor-mediated signaling to inhibit high mobility group box-1 (HMGB1) protein release from *I*/*R* injury [9]. Our study suggested that EA at Neiguan (PC6) has a high consistency with Jiaji (EX-B2), and both groups have steroid hormone to protect the myocardium during *I*/*R*. Huang and colleagues first investigated genome-wide gene expressions and the cardioprotective effects of EA pretreatment at PC6 on myocardial *I*/*R* injury. Their results showed that the Neiguan acupoint specifically regulated cardiac muscle contraction, vascular smooth muscle contraction, hypertrophic cardiomyopathy, oxidative phosphorylation, inflammation and immune response, and apoptosis pathways, thus effectively protecting against *I*/*R* injury in a pretreatment approach. Similar to their results, we observed the activation of functions associated with inflammatory pathways. The overlapping DEGs between the JJ and NG group were enriched at the chemokine signaling pathway and cytokine-cytokine interaction. However, we did not observe enrichment in pathways related to myocardial dysfunction. This may be due to the different reperfusion times in our respective studies [10]. In addition, we found that circadian rhythm gene expression was reduced after electroacupuncture preconditioning at Neiguan. Recent studies suggest that the cardiomyocyte circadian clock directly modulates heart responsiveness of the heart to *I*/*R* and influences insulin-regulated glucose utilization processes [28, 29]. The antiarrhythmic effect of EA pretreatment at Neiguan has been verified [4, 26]. To our knowledge, this is the first study to discover the association between Neiguan acupoints and circadian rhythms. In addition, we also found that circadian entrainment was significantly upregulated. Via re-entrainment, the upregulation of circadian entrainment may allow the reprogramming of a new circadian rhythm adapted to the environment or stress. Although the Neiguan and Jiaji acupoints both revealed significant effects against *I*/*R* injury, they showed different specifically regulated biological processes. EA at Jiaji enriched more BPs than any other preconditioning treatment. The BPs' neurohumoral regulation, metabolic processes, and immunity BPs were markedly increased in the JiaJi group. The most enriched BP in the JiaJi group was oxygen transport, while the most important enriched BP in the Neiguan group was IL-33 signaling. Previous studies have revealed a strong correlation between IL-33 and myocardial fibrosis. IL-33 binds the ST2 receptor (ST2L) on the cardiomyocyte membrane, promoting cell survival and integrity [30]. Our data showed that EA at Neiguan could significantly upregulate the IL-33 signaling pathway.

In the traditional Chinese medicine theory, meridians and acupoints have certain specificity. Previous studies have shown that there was no cardioprotective effect in *I*/*R* rats when EA was performed at a nonacupoint (base of tail) [10]. However, there have been no studies about meridian and acupoint specificity, that is, comparisons of acupoints that are and are not directly involved in the heart. Therefore, “Yanglingquan” (GB34) and “Quchi” (LI11) acupoints were selected as controls. Yanglingquan (GB34) belongs to the gallbladder meridian, which contacts the heart with its divergent meridians. Quchi (LI11) acupoints, which are on the large intestinal meridian, have no relationship with the heart. Previous studies have proven that stimulation at GB34 showed better neuroprotective effects. EA at GB34 promoted the autophagic clearance of *α*-synuclein and reduced oxidative stress in Parkinson's disease (PD) mice [15, 16]. Based on the number of DEGs enriched, the most interesting functional categories were “regulation of nervous system development” and “sex hormone regulation” in the YLQ group. Stimulation at GB34 could increase the production of sex hormones, possibly because it contacted the heart with its divergent meridians. However, this indirect relationship could not reverse *I*/*R* injury. In the clinical practice, LI11 has been used to treat digestive system diseases and hypertension [18]. The antihypertension effect of LI11 occurs by decreasing sympathetic nerve activity, which increases the baroreflex sensitivity in hypertensive rats [1, 17]. Our data suggested that EA pretreatment at LI11 suppressed cell cycle gene expression and activated nervous system signal transduction and gastric acid secretion in *I*/*R* rats. This may be because LI11 belongs to the large intestinal meridian and is involved in the nervous system.

Given the underlying cell response properties, acupuncture studies could not be carried out in vitro. In accordance with the rules of 3R, the sample size chosen was adequate to reveal only preliminary effects of EA at Jiaji (EX-B2) acupoints. The limitation of this study is that we did not further verify the mechanism. Therefore, further studies should address the cardioprotective molecular mechanism of Jiaji (EX-B2) acupoints, possibly indicating new perspectives for clinical practice.

## 5. Conclusions

In conclusion, our results clearly indicated that EA pretreatment at T4-T5 Jiaji (EX-B2) acupoints exerts a cardioprotective effect in *I*/*R* rats by modulating functional gene expressions. Our study provided, for the first time, informative genome-wide profiles of gene expressions after stimulation of acupoints belonging to different meridians in *I*/*R* injury. There is a specific connection between meridians and Zangfu-organs. Stimulation at Jiaji specifically modulated the neural-immune network to protect against *I*/*R* injury. The present study also found a link between EA at Neiguan and circadian rhythm. It seems worthwhile to further investigate the feedback mechanism. Thus, our work highlights that EA at the T4-T5 Jiaji (EX-B2) acupoints could be an appropriate alternative treatment for patients with *I*/*R*.

## Figures and Tables

**Table 1:** Differentially expressed genes across six groups.

DEGs	*I*/*R* vs. Sham	JJ vs. *I*/*R*	NG vs. *I*/*R*	YLQ vs. *I*/*R*	QC vs. *I*/*R*
Upregulated	456	558	520	229	336
Downregulated	115	161	53	361	150
Total	571	719	573	590	486

## Data Availability

The microarray raw data are shown in GEO with the number GSE100499. The data can be obtained from the corresponding author and co-authors on request.
